# Decrease in ovarian reserve through the inhibition of SIRT1-mediated oxidative phosphorylation

**DOI:** 10.18632/aging.203942

**Published:** 2022-03-11

**Authors:** Lu Guo, Xiaocheng Liu, Hua Chen, Weigui Wang, Chao Gu, Bin Li

**Affiliations:** 1 Department of Gynecology, Obstetrics and Gynecology Hospital of Fudan University, Shanghai 200011, China; 2 Shanghai Key Laboratory of Female Reproductive Endocrine Related Diseases, Shanghai 200090, China

**Keywords:** ovarian reserve, premature ovarian insufficiency, oxidative stress, mouse model, 3-nitropropionic acid

## Abstract

Objective: To establish an oxidative stress-induced model of premature ovarian insufficiency (POI) and to explore the effect of SIRT1 and mitochondrial oxidative phosphorylation on the ovarian reserve.

Methods: Mice were treated with intraperitoneal injections of 3-nitropropionic acid (3-NPA) at different doses and for different time periods to induce a model of POI. Subsequently, the efficiency of each regimen was evaluated. The expression of SIRT1 in ovarian tissue was examined. Then, SIRT1 was knocked down in human luteinized granulosa cells (GCs), and its function and related receptor and gene expression were examined. Finally, a SIRT1 antagonist and agonist were used to explore the effects of SIRT1 on ovarian function *in vivo* and on the change in mitochondrial oxidative phosphorylation complexes (OXPHOS).

Results: Decreases in ovarian reserve were successfully induced through the intraperitoneal injection of 40 mg/kg 3-NPA for 3 weeks, and SIRT1 was down-regulated in the model group. The knockdown of SIRT1 impaired the estrogen synthesis capacity of human GCs and decreased the expression of related genes. 3-NPA and SIRT1 antagonist Ex-527 decreased ovarian function and inhibited OXPHOS. In contrast, the SIRT1 agonist resveratrol promoted the recovery of ovarian function in the model group and improved OXPHOS. Additionally, P53, CASPASE 3, and BAX were down-regulated and BCL2 was up-regulated in the 3-NPA and Ex-527 groups; opposite trends were observed after resveratrol treatment.

Conclusions: The intraperitoneal injection of 40 mg/kg 3-NPA for 3 weeks could effectively induce POI. The increase in oxidative stress inhibited SRIT1 and mitochondrial oxidative phosphorylation, inducing follicular apoptosis.

## INTRODUCTION

With the delay in female childbearing age and the initiation of the three-child policy, more and more attention has been paid to problems associated with ovarian aging. The mechanisms involved in ovarian aging are not fully understood. Premature ovarian insufficiency (POI) is a common cause of early menopause, affecting about 1-2% of women of childbearing age [1]. Elevated levels of oxidative stress are considered to be the main cause of decline in oocyte quality [2]. Oxidative stress, which is related to mitochondrial dysfunction — a cause of ovarian aging — is considered as an important contributor to premature ovarian insufficiency (POI) [3]. Because existing POI models do not consider the relationship between ovarian reserve and oxidative stress, it is of great significance to establish a POI model of oxidative stress. 3-nitropropionic acid (3-NPA), a mitochondrial complex II inhibitor, is mainly used in neurological research. Its effect on ovarian function has not been reported and requires further exploration [4]. SIRT1 was reported to have a strong correlation with ovarian reserve and could be a marker of ovarian aging [5]. SIRT1-overexpressing mice were found to have more primordial follicles and fewer atretic follicles [6]. Decreased SIRT1 levels were shown to cause mitochondrial dysfunction by increasing oxidative stress and DNA damage, leading to infertility [7]. The up-regulation or overexpression of SIRT1 could cause the activation of primordial follicles, whereas SIRT1 knockdown in oocytes or granulosa cells suppressed this activation [8]. However, the relationship between SIRT1 and mitochondrial oxidative phosphorylation in POI models has so far remained unclear. Thus, this study aimed to establish a 3-NPA-induced model of POI and to explore the role of SIRT1 and oxidative phosphorylation in this process.

## RESULTS

### 3-NPA treatment induced POI-like manifestations

**Figure 1:**
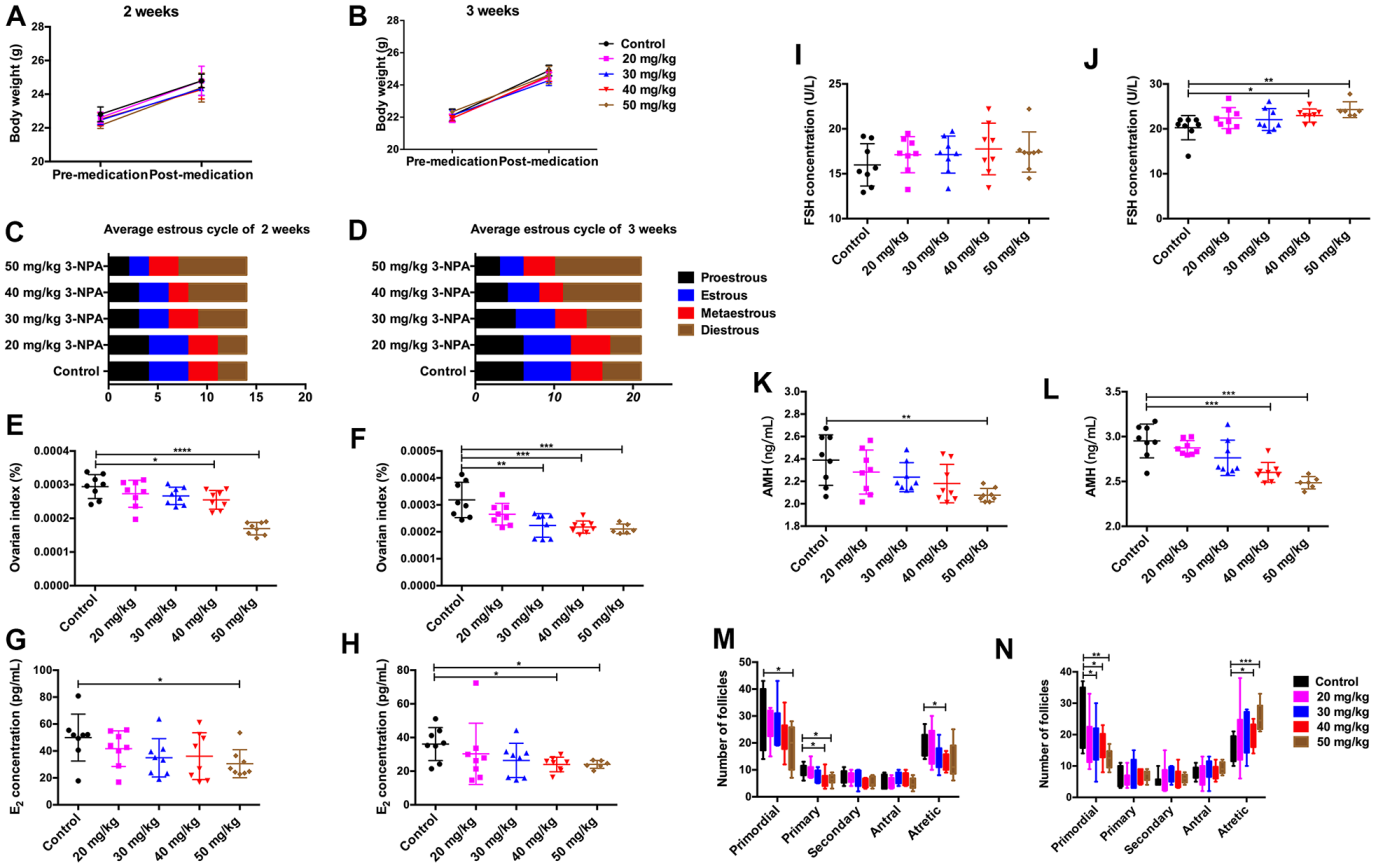
**Comparison of clinical manifestations at different doses of 3-NPA.** (**A**, **B**) 3-NPA had no significant effects on body weight. (**C**) 3-NPA could prolong the mean length of the diestrus phase and decrease the mean length of the estrous phase after two weeks of treatment, especially when used at a dose of 50 mg/kg. (**D**) 3-NPA could prolong the mean length of the diestrus phase and decrease the mean length of the estrous phase after three weeks of treatment, especially when used at a dose of 40 mg/kg and 50 mg/kg. (**E**, **F**) 3-NPA reduced the ovarian index, especially when used at a dose of 40 mg/kg and 50 mg/kg. (**G**, **H**) Serum E_2_ concentration in different groups; mice treated with 40 mg/kg 3-NPA for 3 weeks and those treated with 50 mg/kg 3-NPA for 2 or 3 weeks showed a significant decrease. (**I**, **J**) Serum FSH concentration in the different groups; mice treated with 40 mg/kg or 50 mg/kg 3-NPA for 3 weeks showed a significant increase. (**K**, **L**) Serum AMH concentration in different groups; mice treated with 40 mg/kg 3-NPA for 3 weeks and those treated with 50 mg/kg 3-NPA for 2 or 3 weeks showed a significant decrease. (**M**) In the mice treated with 50 mg/kg 3-NPA for 2 weeks, the number of primordial follicles decreased. (**N**) In the mice treated with 40 mg/kg or 50 mg/kg 3-NPA for 3 weeks, the number of primordial follicles decreased, the number of atretic follicles increased, and the number of growing follicles did not change significantly. (N=8 in all assays; *P<0.05, **P<0.01, and ***P<0.001 compared with the control group, NS: none significant).

**Figure 2:**
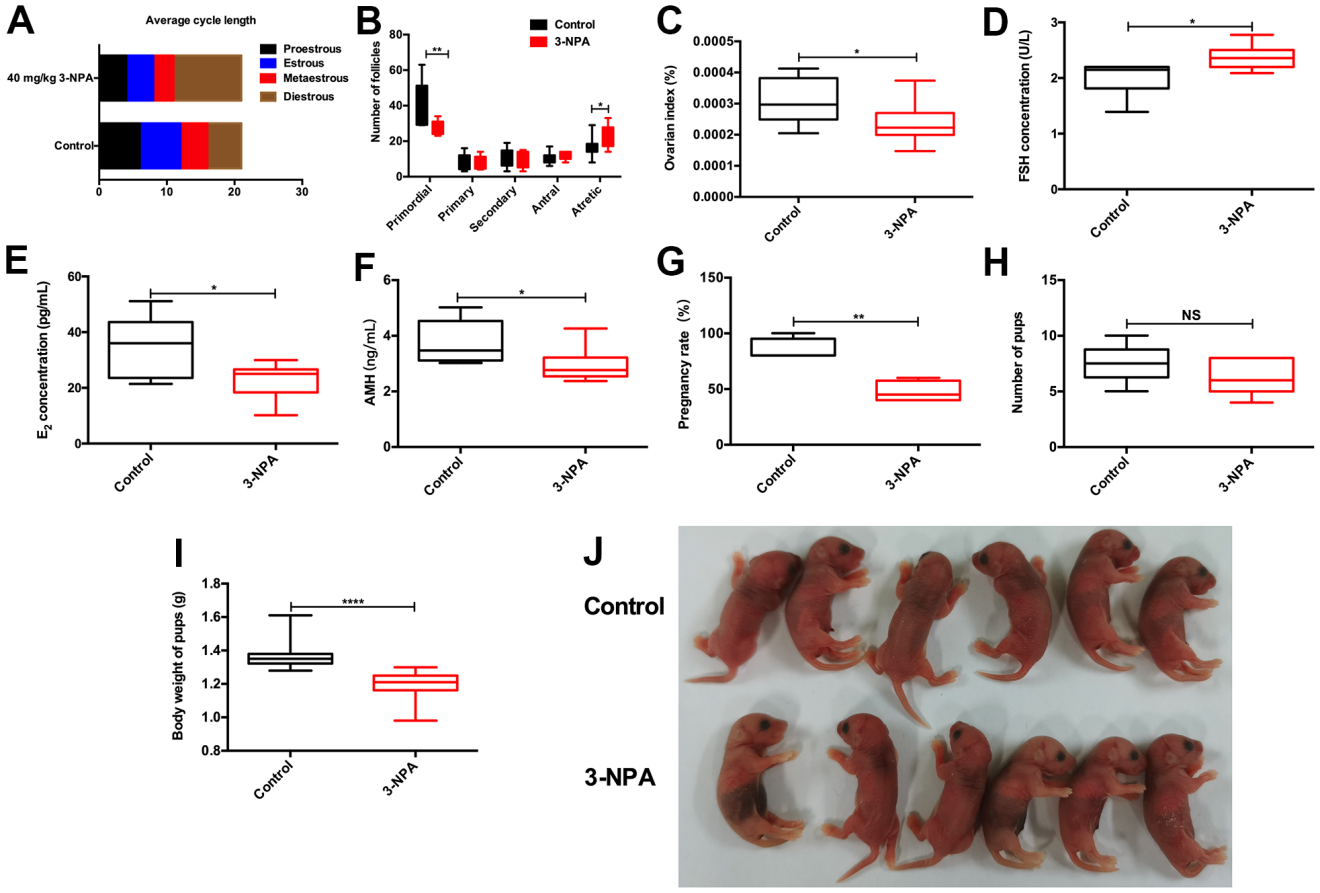
**40 mg/kg 3-NPA significantly induced manifestations similar to those of POI.** (**A**) 40 mg/kg 3-NPA could prolong the mean length of the diestrus phase and decrease the mean length of the estrous phase after three weeks of treatment. (**B**) 40 mg/kg 3-NPA could decrease the number of primordial follicles and increase that of atretic follicles. (**C**–**F**) 40mg/kg 3-NPA decreased the ovarian index (**C**), increased serum FSH levels (**D**), decreased serum E_2_ levels (**E**), decreased serum AMH levels (**F**), and decreased mouse fertility (**G**). (**H**) Although the 40 mg/kg 3-NPA group had fewer pups, the difference was not significant. (**I**, **J**) The offspring of the mice treated with 40 mg/kg 3-NPA showed lower weight than the control group (N=8 in all assays; *P<0.05, **P<0.01).

**Figure 3:**
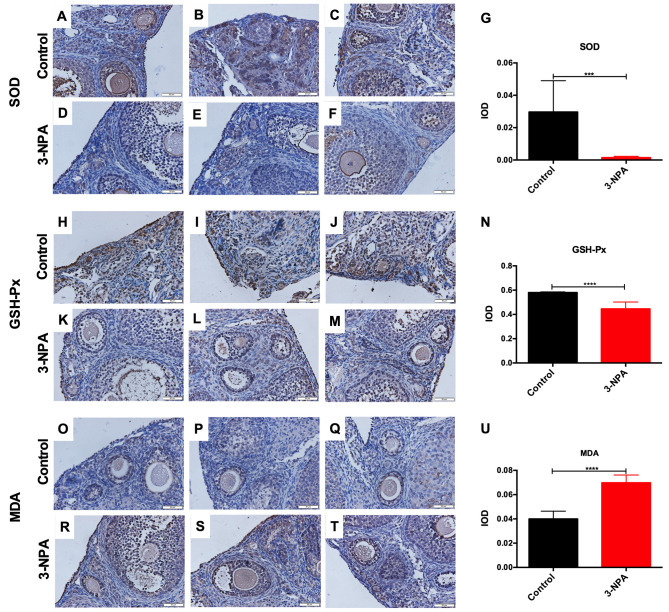
**Comparison of ovarian oxidative stress-related changes between the control and 3-NPA groups.** (**A**–**U**) SOD expression was lower (**A**–**G**), GSH-Px expression was lower (**H**–**N**), and MDA expression was higher (**O**–**U**) in GCs from 3-NPA mice than in those from control mice (Triplicates slides in each group, N=8 in all assays; *P<0.05, **P<0.01, ***P<0.001, ****P<0.0001).

No significant differences on body weight were observed among the different 3-NPA treatment groups. 3-NPA had a strong influence on the estrous cycle of female mice. It significantly prolonged the diestrus phase while decreasing the estrous phase, especially when used at a dose of 50 mg/kg. In addition, the 50 mg/kg group showed obviously shrunken ovaries, consistent with lower ovary weight, although their overall body weight showed no significant changes. Two-week treatment with 50 mg/kg 3-NPA decreased serum E_2_ and AMH levels, while three-week treatment with both 40 mg/kg and 50 mg/kg 3-NPA increased serum FSH and decreased serum E_2_ and AMH levels. In addition, two-week treatment with 50 mg/kg 3-NPA and three-week treatment with both 40 mg/kg as well as 50 mg/kg 3-NPA decreased the number of primordial follicles and increased the number of atretic follicles. However, the treatments had no significant effect on the number of growing follicles (Figure 1). In the 50 mg/kg group, 25% of mice died and the remaining mice were included in statistical analysis. Hence, 40 mg/kg 3-NPA treatment for 3 weeks seemed to be the most appropriate treatment strategy because it prolonged the diestrus phase; shortened the estrous phase; and decreased the number of primordial follicles, ovarian index, serum E_2_ levels, AMH levels, and fertility. An increased number of atretic follicles and serum FSH levels were also observed. Further experiments revealed that 40 mg/kg 3-NPA treatment for 3 weeks reduced the body weight of pups, indicating that this treatment could successfully decrease ovarian reserve and fertility and induce POI (Figure 2). In addition, ovarian GSH-Px and SOD expression were also decreased by this treatment and MDA was increased (Figure 3). Further, the safety of 3-NPA was also evaluated. In addition to body weight, mouse creatine, glutamic pyruvic transaminase (GPT), and glutamic oxaloacetic transaminase (GOT) levels also remained stable. These levels were similar between the 40 mg/kg 3-NPA group and the control group (Supplementary Figure 1).

### SIRT1 influenced the function of human luteinized granulosa cells (GCs)

**Figure 4:**
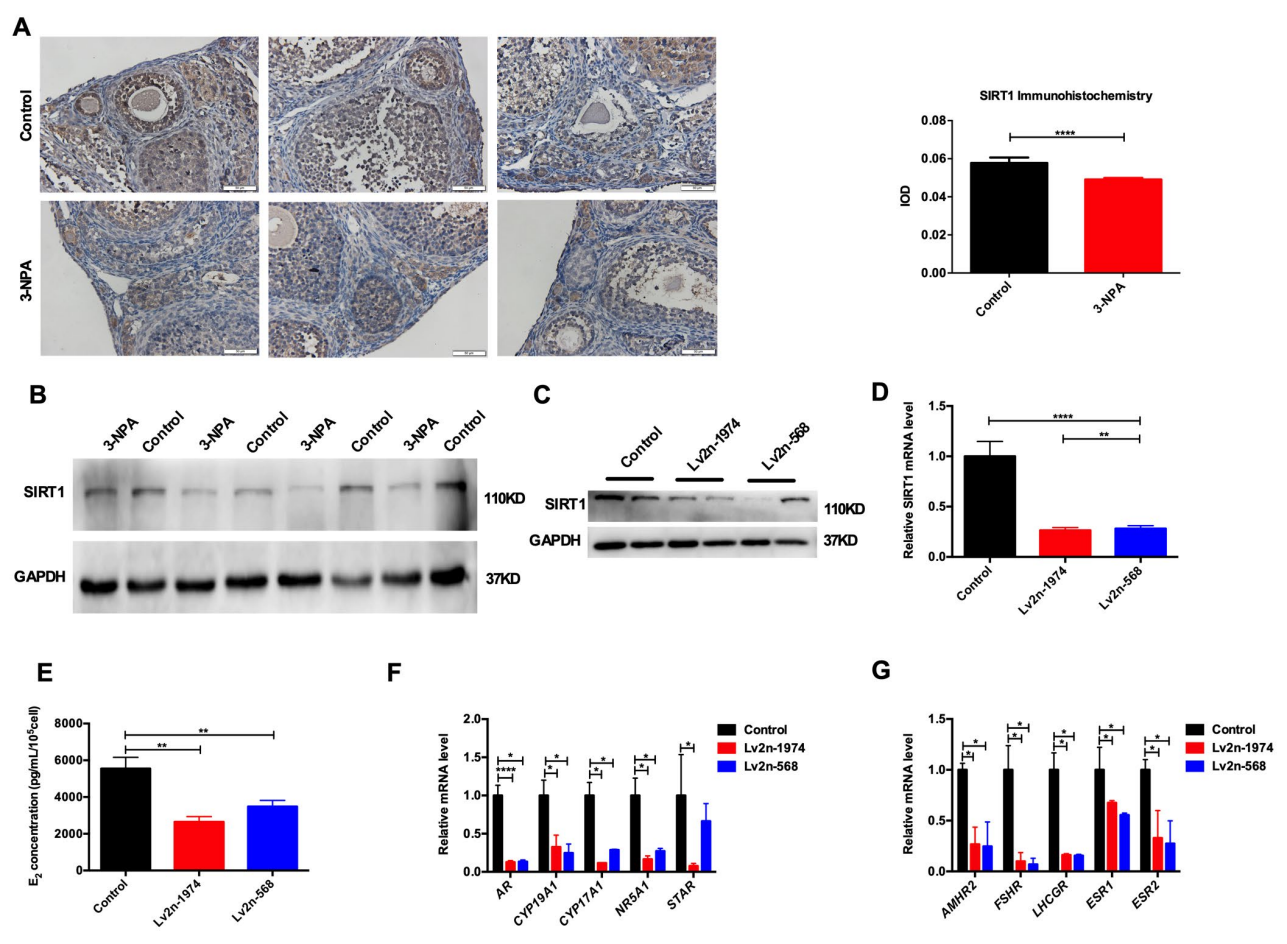
**SIRT1 is involved in the function of human GCs.** (**A**) SIRT1 was downregulated in the 3-NPA model group in immunohistochemistry (Triplicates slides in each group). (**B**) SIRT1 was downregulated in the 3-NPA model group in western blot (Quadruplicates samples in each group). (**C**, **D**) SIRT1 was knocked down using a lentivirus. (**E**) Estrogen synthesis decreased after SIRT1 knockdown. (**F**) Genes related to estrogen synthesis were down-regulated after SIRT1 knockdown. (**G**) Functional receptors were down-regulated in GCs after SIRT1 knockdown (*P<0.05, **P<0.01, ***P<0.001, ****P<0.0001).

Compared with that in the control group, the expression of SIRT1 in ovarian tissue in the POI mouse model was lower (Figure 4A, 4B). Because GCs are important ovarian stromal cells, we examined whether SIRT1 influenced the function of GCs. We knocked down SIRT1 using a lentivirus and verified transfection efficiency using RT-PCR and western blotting (Figure 4C, 4D). The synthesis and secretion of E_2_ in the lv2n-568 and lv2n-1974 intervention group was significantly lower. The expression of genes involved in estrogen synthesis, such as *AR*, *CYP19A1*, *CYP17A1*, *NR5A1*, and *STAR* and that of functional receptors *FSHR*, *AMHR2*, *LHCGR*, *ESR1*, and *ESR2* showed a decrease after SIRT1 knockdown (Figure 4E–4G), suggesting that SIRT1 may be involved in regulating hormone synthesis capacity and receptor expression in human GCs.

### SIRT1 influenced ovarian reserve in mice

**Figure 5:**
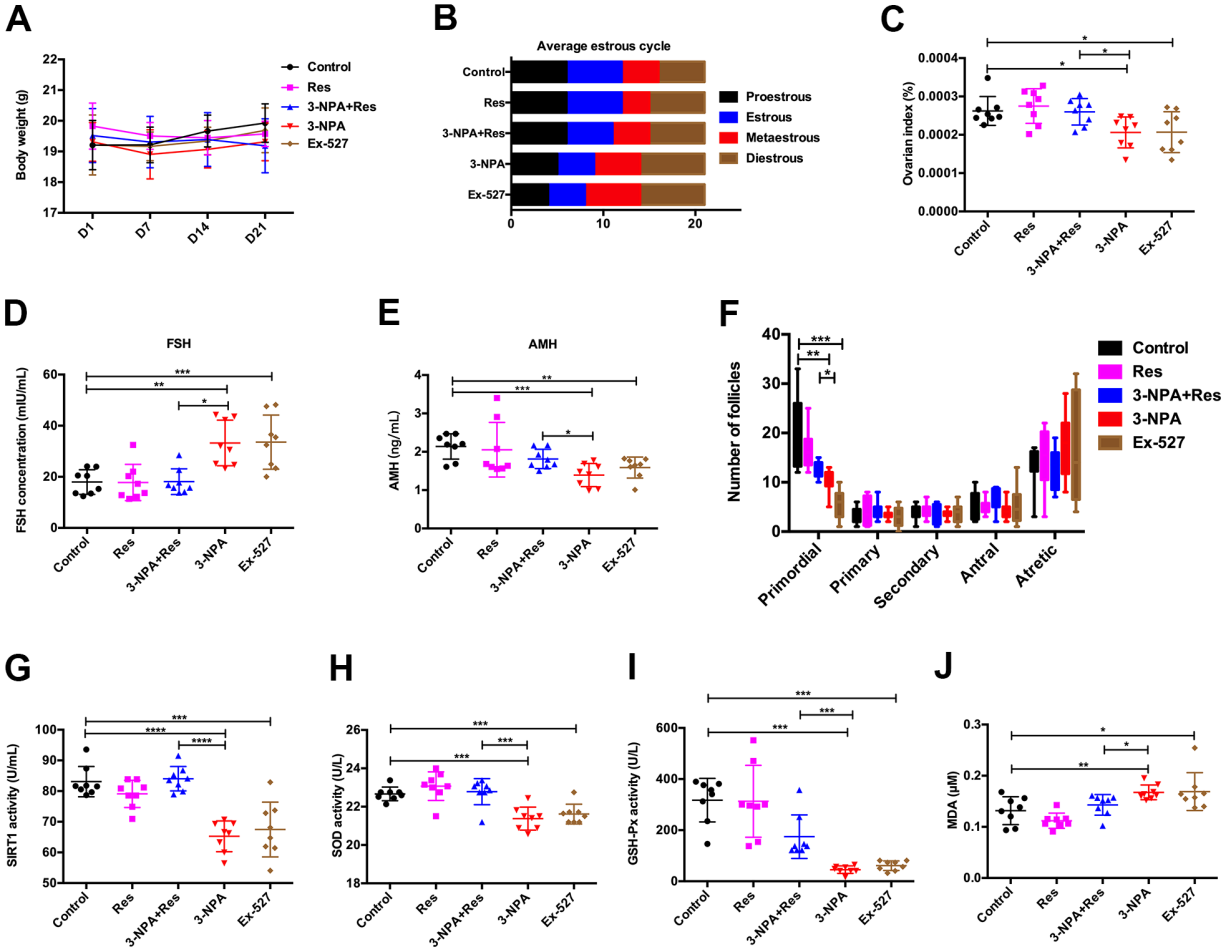
**Res improves ovarian function by activating SIRT1.** (**A**) No changes in body weight. (**B**) Ex-527 and 3-NPA prolonged the mean length of the diestrus phase and decreased the mean length of the estrous phase, while Res had the opposite effect. (**C**) Ex-527 and 3-NPA decreased the ovarian index and Res increased the ovarian index. (**D**) Ex-527 and 3-NPA increased serum FSH levels, and Res decreased these levels. (**E**) Ex-527 and 3-NPA decreased serum AMH levels and Res increased these levels. (**F**) Ex-527 and 3-NPA decreased the number of primordial follicles. (**G**) Ex-527 and 3-NPA decreased SIRT1 activity, while Res increased it. (**H**, **I**) Ex-527 and 3-NPA could decrease SOD (**H**) and GSH-Px (**I**) activity, while Res could increase SOD (**H**) and GSH-Px (**I**) activity. (**J**) Ex-527 and 3-NPA increased MDA activity, while Res decreased this activity (N=8 in all assays; *P<0.05, **P<0.01, ***P<0.001, ****P<0.0001).

To further verify the role of SIRT1 in the development of POI, intraperitoneal injections of the SIRT1 inhibitor Ex-527 and agonist Resveratrol (Res) were administered. Body weight did not differ among the five groups of mice. In the Ex-527 and 3-NPA groups, the average diestrus period was prolonged and the average estrous period was shortened. Furthermore, these groups showed a decrease in the ovarian index, AMH levels, and the number of primordial follicles along with an increase in serum FSH levels. In contrast, in the 3-NPA+Res group, the average diestrus period was shortened and the average estrous period was prolonged. This group showed an increase in the ovarian index, AMH levels, and the number of primordial follicles along with a decrease in serum FSH levels, indicating that Res could improve ovarian reserve. Moreover, the activities of serum SIRT1, SOD, and GSH-Px were reduced in the Ex-527 and 3-NPA groups and the activity of MDA was increased; these effects were reversed in the 3-NPA+Res group. The above results indicated that SIRT1 inhibition may decrease the ovarian reserve, inducing a POI phenotype, and that SIRT1 agonists can improve the ovarian reserve, relieving POI symptoms (Figure 5).

### Mitochondrial oxidative phosphorylation induced the apoptosis that resulted in POI

Mitochondria are the main organelles where oxidative stress occurs, and their role in the occurrence of POI remains to be explored. Apoptosis, nuclear pyknosis, chromatin agglutination and marginalization, and cytoplasmic vacuolation were obvious in GCs from the 3-NPA and Ex-527 groups under electron microscopy. Moreover, swollen mitochondria, decreased matrix density, and marginally shifted cristae were also detected. In contrast, amelioration of mitochondrial swelling was observed in the 3-NPA+Res group.

**Figure 6:**
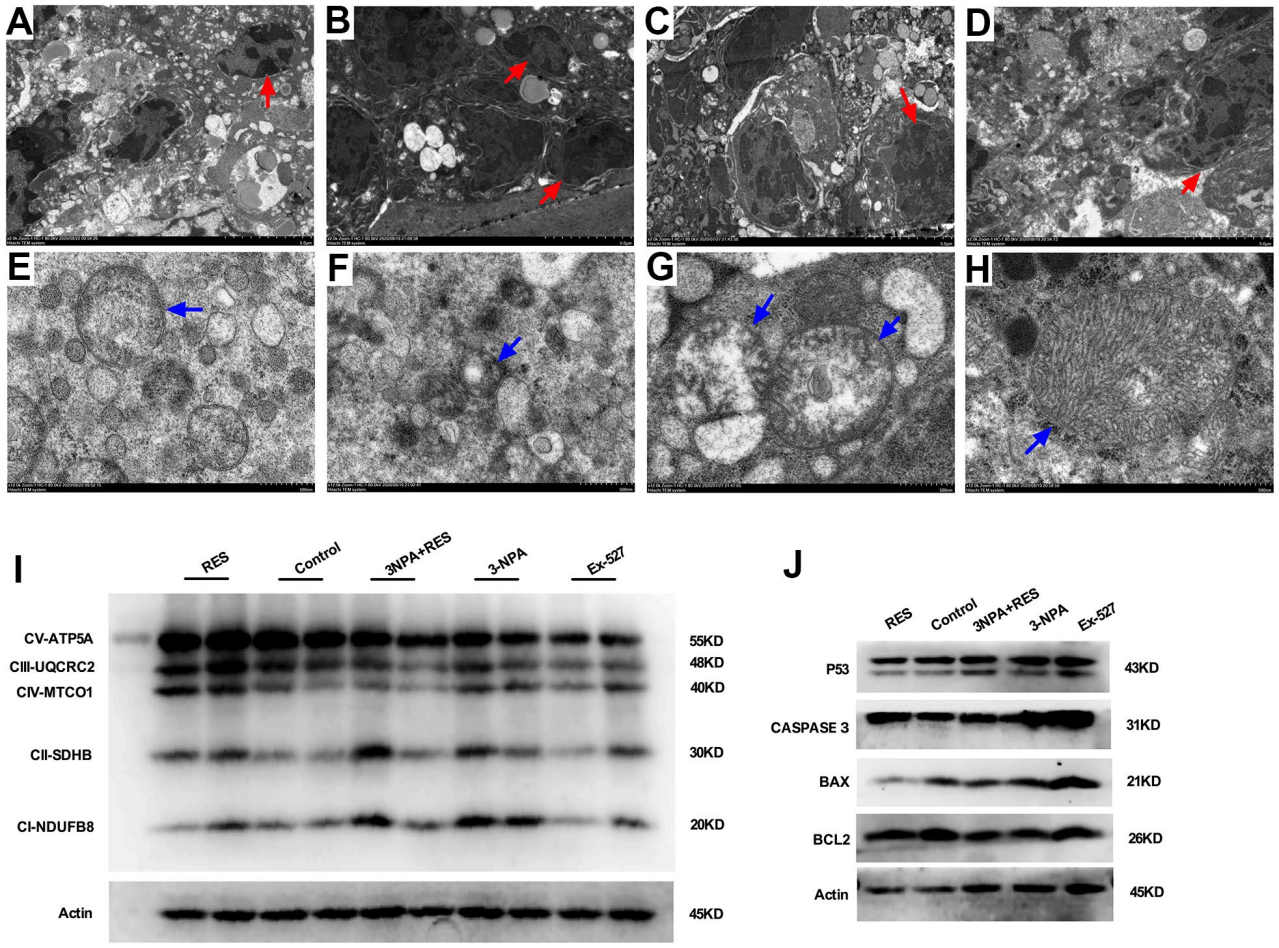
**Mitochondrial oxidative phosphorylation-induced apoptosis may result in oxidative stress-induced POI.** (**A**) Granulosa cells in ovarian tissue from the control group. (**B**) In the 3-NPA model group, GCs apoptosis and nuclear pyknosis were obvious, the chromatin margin was set, organelle structure was destroyed, and empty shots appeared. (**C**) Obvious granulocyte apoptosis was observed in ovarian tissue from mice in the Ex-527 intervention group; chromatin agglutination and vacuoles were also observed. (**D**) Ovarian granulocyte apoptosis was significantly lower in the resveratrol intervention group than in the model group. (**E**) Mitochondrial morphology in ovarian tissue from mice in the control group was largely normal, and the number of mitochondria was high. (**F**) In the 3-NPA group, mitochondrial morphology in ovarian tissue was destroyed, stromal edema was obvious, and the endoplasmic reticulum was dilated and damaged. (**G**) Ovarian mitochondria in mice from the Ex-527 intervention group were obviously swollen and cavitated. (**H**) Ovarian mitochondria in the resveratrol intervention group showed significantly improved morphology; the mitochondrial crest was clearly visible, and the mitochondrial matrix was uniform. (**I**) OXPHOS expression was down-regulated in the Ex-527 and 3-NPA groups, while it was up-regulated in the Res group. (**J**) P53, BAX, and CASPASE3 were up-regulated in the Ex-527 and 3-NPA groups and down-regulated in the Res group. BCL2 was down-regulated in the Ex-527 and 3-NPA groups, while it was up-regulated in the Res group. Red arrow: granular nucleus; Blue arrow: mitochondria.

Further, mitochondrial oxidative phosphorylation complexes (OXPHOS) showed lower expression in the 3-NPA and Ex-527 groups and higher expression in the 3-NPA+Res group than in the control group. Apoptosis was obvious in the 3-NPA and Ex-527 groups under electron microscopy, while reduced apoptosis was noted in the 3-NPA+Res group. Further experiments showed that P53, CASPASE 3, and BAX were up-regulated and that BCL2 was down-regulated in the 3-NPA and Ex-527 groups, while the 3-NPA+Res group showed P53, CASPASE 3, and BAX down-regulation and BCL2 up-regulation. These results indicated that SIRT1 can increase ovarian reserve by influencing the structure and function of mitochondria, inhibiting follicular apoptosis, and increasing the number of primordial follicles (Figure 6).

## DISCUSSION

ROS has been known to contribute to the formation of primordial follicles and follicular development, maturation, and atresia [9, 10]. However, the mechanism by which oxidative stress reduces ovarian reserve has so far remained unclear. 3-NPA could trigger mitochondrial ROS production and accumulation, leading to mitochondrial DNA damage and affecting mitochondrial function. Accordingly, a murine model of POI was created in the present study. It was confirmed that 3-NPA treatment could simulate the clinical manifestations of POI, indicating that oxidative stress may be the underlying etiology in cases of decreased ovarian reserve. H_2_O_2_ was the agent most commonly used to establish cellular models of oxidative stress.

However, research in animal experiments was limited owing to the instability of this compound. Compared to existing POI models, the gene knockout model was expensive. The enzyme defect model, immune model, chemotherapy drug-based model, and radiation model all showed a variety of defects, and their correlation with oxidative stress was not well-documented [11, 12]. Thus, our model could be of great significance for exploring the relationship between oxidative stress and POI.

Oxidative stress has been suggested to induce granulosa cell apoptosis, contributing to follicular atresia. SIRT1 is known to act as an energy sensor, and its suppression was found to result in accelerated primordial follicle loss and follicle dormancy, which affected the fate of primordial follicles [13]. miR-181a increased oxidative stress-induced FoxO1 acetylation and promoted the apoptosis of mouse ovarian granulosa cells via SIRT1 downregulation both *in vitro* and *in vivo* [14]. The SIRT1 activator, resveratrol, and the SIRT1 inhibitor, Ex527, could reduce or elevate the H_2_O_2_-induced apoptosis of human granulosa COV434 cells through the modulation of the P53/SIRT1 axis, respectively [15]. However, none of previous studies uncovered the correlation between SIRT1 and POI. Consistent with previous reports, in this study, a weakened estrogen synthesis ability and the reduced expression of related genes were observed after inhibition in human GCs. Moreover, the ovarian SIRT1 expression was found to be decreased in the POI model. The SIRT1 inhibitor Ex-527 impaired ovarian function, whereas Res improved ovarian function in the POI model, suggesting that SIRT1 may be involved in preventing oxidative stress-induced POI.

Oxidative stress can negatively affect mitochondrial functions in GCs during follicular development, and antioxidant therapy can ameliorate oxidative stress and mtDNA damage [16]. Mitochondrial gene expression and protein synthesis were found to play a primary role in ATP production, which is necessary for GC proliferation and differentiation during follicular development [17]. In addition to genetic mutations in mitochondria, increased ROS and decreased oxidative phosphorylation activity and ATP production in oocytes were also associated with POI [18]. Furthermore, mitochondria played an important role in the biosynthesis of steroid hormones [19]. Loss of SIRT1 in oocytes affected ovarian reserve, follicular maturation, mitochondrial abundance, and fertility, resulting in premature aging [20]. Our study revealed that the inhibition of SIRT1 impairs mitochondrial function and mitochondrial oxidative phosphorylation, stimulating the follicular apoptosis, while the overexpression of SIRT1 promotes the recovery of mitochondrial function and decreases the follicular atresia. We concluded that oxidative stress inhibited SIRT1, leading to impaired mitochondrial oxidative phosphorylation, increased apoptosis of follicles, depletion of primordial follicles, and decrease in ovarian reserve.

In conclusion, our study demonstrated for the first time that 3-NPA could induce POI manifestations successfully through oxidative stress. SIRT1 played a role in this process by inhibiting oxidative phosphorylation and inducing follicular apoptosis. However, there are some limitations to this study. Oxidative stress occurs in mitochondria, and SIRT1 is mainly expressed in the nucleus. The interactions among oxidative stress, SIRT1, and the mitochondria need to be studied further. In addition, it was found that Res also promotes the expression of SIRT3, which is closely involved in oxidative stress. Further experiments are required to exclude the influence of SIRT3. Nevertheless, the molecular mechanisms of POI uncovered in this study could help in optimizing treatment and slowing down ovarian aging. This could have a great impact on improvements in life quality and pregnancy rates among women.

## MATERIALS AND METHODS

### Establishment of the mouse model

Six-week-old female C57BL/6 mice were obtained from the Jie Sijie Animal Center (Shanghai, China), and the study was approved by the Animal Experimental Ethical Committee of Fudan University. Eighty mice were randomly divided into 10 groups (N=8 per group). The first five groups received intraperitoneal injections of the same volume of sterile water and 20 mg/kg, 30 mg/kg, 40 mg/kg, and 50 mg/kg 3-NPA (Sigma-Aldrich) every morning for 14 days and the last five groups received these intraperitoneal injections for 21 days. The body weight of mice was recorded before and after 3-NPA administration. Vaginal smears were obtained each morning to monitor the estrous cycles of mice [21]. Blood was collected before sacrifice, and centrifuged at 295 x*g* for 10 min, and stored at -80° C for subsequent evaluation. Ovaries were removed and weighed immediately. The ovarian index was calculated as the ratio of ovary weight to the weight of the mouse. Then, the ovaries were immediately fixed in 4% paraformaldehyde, embedded in paraffin, and sectioned into 5-μm-thick slices. The sections were stained with hematoxylin and eosin, and follicles were counted under a microscope (Olympus), as previously described [22, 23].

### Mating experiments

After intraperitoneal injection, 5 female mice from each group were caged with male C57BL/6 mice at a 2:1 ratio for 2 weeks. Once mating was confirmed through the presence of fertilization plugs, the females were separated [24]. The rate of pregnancy, number of pups, and weight of pups were recorded.

### Enzyme-linked immunosorbent assay (ELISA)

Serum FSH, E_2_, AMH, SOD, GSH-Px, MDA, and SIRT1 activities were measured using ELISA based on the manufacturer’s instructions. Mouse FSH ELISA kits were purchased from CUSABIO (CSB-E06871; assay range, 4–140 mIU/mL). Mouse E_2_ ELISA kits were purchased from LDN (FRE-2000; assay range, 25–2000 pg/mL). AMH ELISA kits were purchased from Aviva Systems Biology (OKEH00320; assay range, 0.156–10 ng/mL). GSH-Px kits were purchased from Bioassay Systems (EGPX-100, America; assay range, 40–800 U/L). MDA kits were purchased from Bioassay Systems (DTBA-100, America; assay range, 0.1–1.5 μM). SOD kits were purchased from DOJINDO (S311, Japan; assay range, 0–100%). SIRT1 ELISA kits were purchased from Jianglai Biology (JL20291; assay range, 1–80 U/mL).

### Immunohistochemistry

After deparaffinization, antigen retrieval was performed by microwaving the sections with citrate buffer (pH 6.0). Endogenous peroxidase activity was inhibited using 3% H_2_O_2_ for 15 min, and non-specific binding was blocked with 10% normal goat serum (WGAR1009-5, Servicebio). All sections were incubated with primary antibodies against SOD (1:500, Santa Cruz), GSH-Px (1:500, Abcam), and MDA (5 μg/mL, Abcam) overnight at 4° C. Biotinylated secondary antibodies (1:500, Servicebio) were added and incubated with the sections for 60 min. Their presence was visualized using a diaminobenzidine (DAB) reaction. Finally, the slides were counterstained with hematoxylin. Image-Pro Plus 6.0 (Media Cybernetics, Inc., MD, USA) was used to measure the integrated optical density (IOD) and area of staining.

### Western blot

Protein concentration was determined using BCA kits (Beyotime Biotechnology). Subsequently, 25 μg of protein was electrotransferred to a polyvinylidene fluoride membrane (Millipore). The membrane was incubated overnight at 4° C with primary antibody against SIRT1 (1:2000, ab110304, Abcam), OXPHOS (1:500, ab110413, Abcam), P53 (1:1000, A5761, ABclonal), CASPASE 3 (1:1000, ab214430, Abcam), BCL2 (1:1000, ab182858, Abcam), BAX (1:1000, ab182733, Abcam). This was followed by secondary antibody incubation (1:1000, Bioss) for 1 hour the next day. Antibody binding was detected using the ECL Western Blotting Substrate (Millipore, WBKLS0500).

### Culture of human luteinized granulosa cells (GCs)

Human luteinized GCs were collected from patients undergoing intracytoplasmic single sperm microinjection (ICSI) in our reproductive center after approval from the Ethics Committee of Obstetrics and Gynecology Hospital of Fudan University (approval number: 2019-106). All these patients were fertile and required this treatment owing to male factors. The GCs were cultured for 24 h in DMEM/F12 medium containing 15% fetal bovine serum (Gibco) without phenol red and inoculated in six-well plates for subsequent experiments [25, 26].

### Cell transfection

Human GCs were seeded into a six-well plate and incubated overnight. They were then transfected with lentiviral vectors (lv2n-SIRT1-homo-568, lv2n-SIRT1-homo-1974) purchased from GenePharma (Supplementary Table 1). Polybrene (5 μg/μL, GenePharma, Shanghai, China) was added to the transfected cells along with the aforementioned lentivirus. After 48 h of transduction, transfection efficiency was verified.

### Reverse transcription–quantitative polymerase chain reaction (RT–qPCR)

Total RNA was extracted using the TRIzol method (Life technologies, USA) and then reverse transcribed into cDNA using the Prime Script™ RT Master Mix kit (Takara, Japan). mRNA levels were measured using a SYBR Premix Ex Taq kit (Takara, Japan) and normalized based on glyceraldehyde-3-phosphate dehydrogenase (*GAPDH*) levels. All data were calculated using the 2^-ΔΔCT^ method. The primers used are listed in Supplementary Table 2.

### Grouping and treatment of mice

Forty six-week-old female C57BL/6 mice were randomly divided into 5 groups, each with 8 mice: (a) Control group: mice received 0.5% methyl cellulose orally and received intraperitoneal injections of saline; (b) Res group: mice received 40 mg/kg Res (MedChemExpress) orally and received intraperitoneal injections of saline [27]; (c) 3-NPA group: mice received 0.5% methyl cellulose orally and received intraperitoneal injections of 40 mg/kg 3-NPA; (d) 3-NPA+Res group: mice received 40 mg/kg Res orally and received intraperitoneal injections of 40 mg/kg 3-NPA; (e) Ex-527 group: mice received 0.5% methyl cellulose orally and received intraperitoneal injections of 2.5 mg/kg Ex-527 (MedChemExpress) [28]. All groups received the same volume of treatment agents for 21 days.

### Electron microscopy

Ovarian tissues were transferred to an EP tube containing fresh TEM fixative and fixed at 4° C for preservation. The tissue was then washed thrice using phosphate buffer solution, fixed with 1% OsO_4_ for 2 h, and dehydrated with an ethanol gradient. Finally, the tissue was soaked in epoxy resin, embedded, and sectioned ultra-thin (70 nm). The sections were stained with uranium and observed under a transmission electron microscope (HT7800, HITACHI).

### Statistical analysis

All data were expressed as mean ± standard error of mean (SEM). If the data conformed to normal distribution, two-tailed Student’s t-tests were used; otherwise, Mann-Whitney tests were used. GraphPad Prism version 6.0 software was used to analyze experimental data, and *P* < 0.05 was considered statistically significant.

## Supplementary Material

Supplementary Figure 1

Supplementary Tables
