# Supplementary material for: Decrease in ovarian reserve through the inhibition of SIRT1-mediated oxidative phosphorylation
Source: Aging (Albany NY). 2022 Mar 11;14(5):2335–47. doi: 10.18632/aging.203942 (PMC8954953; doi:10.18632/aging.203942)
Supplement: Supplementary Figure 1 [file aging-14-203942-s001.pdf]

SUPPLEMENTARY FIGURE

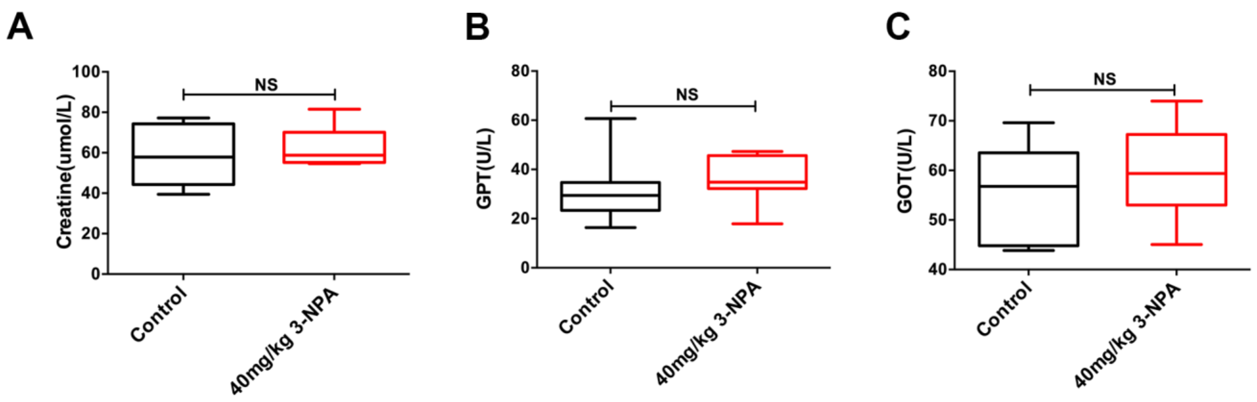

Supplementary Figure 1. 40 mg/kg 3-NPA showed no liver and kidney toxicity. (**A**–**C**) 40 mg/kg 3-NPA had no effect on mouse creatine, GPT and GOT (N=8 in all assays; NS: none significant).
