# Supplementary material for: Decrease in ovarian reserve through the inhibition of SIRT1-mediated oxidative phosphorylation
Source: Aging (Albany NY). 2022 Mar 11;14(5):2335–47. doi: 10.18632/aging.203942 (PMC8954953; doi:10.18632/aging.203942)
Supplement: Supplementary Tables [file aging-14-203942-s002.pdf]

SUPPLEMENTARY TABLES

Supplementary Table 1. Lentiviral sequences.

Group	Target Sequence (5'-3')
LV3NC	TTCTCCGAACGTGTCACGT
Lv2n-SIRT1-homo-1974	GCTTGATGGTAATCAGTATCT
Lv2n-SIRT1-homo-568	GCCTCACATGCAAGCTCTAGT

Supplementary Table 2. Primer sequences.

Gene	(Refseq No.)	Forward (5'-3')	Reverse (5'-3')
GAPDH	NM_002046	ATTTGGTCGTATTGGGCGCC	ACCTCAACTACATGGTTTAC
SIRT1	NM_012238	CATGCCAGAGTCCAAGTTTAG	CATCTCCATCAGTCCCAAAT
AMHR2	NM_020547	GAAAACTGGTTGCCATCAAG	CCACATTGTCCGATTTATCACT
FSHR	NM_000145	GCAGAGCCAGGATTAGGTAT	TTGTAATGCGTCAGTGTGGA
LHCGR	NM_000233	CCATCACCTATGCTATTCAC	TTGGAGGATGGCTCTTTTCT
ESR1	NM_000125	CTCTCTTTCCCCCTTGCTAT	GGTTGGGGCTCAGATAACTC
ESR2	NM_001437	CCATCGCCAGTTATCACATC	GTTCAAAGAGGGATGCTCAC
AR	NM_000044	AAACTCTCACTGCCACTACC	TATGTCCTCCCTTCAGTGTT
CYP17A1	NM_000102	TGTCATCTCCTTGATCTGCTT	ACAACCTGAGCAAAGACAGC
CYP19A1	NM_000103	CAGAAGATACACGACTTGTC	AGCATTTCTCATCAGTAGTT
STAR	NM_000349	TGTACCCACCTAAAACCATC	AGAAGTCTTGCTTTATGGGC
NR5A1	NM_004959	GCCTCAAGTTCATCATCCTC	GCTTGACTACACCCTGTGCC
